# Effect of Crude Extract from the Sea Anemone *Bunodeopsis globulifera* on Voltage-Gated Ion Channels from Central and Peripheral Murine Nervous Systems

**DOI:** 10.3390/ph17081006

**Published:** 2024-07-30

**Authors:** Aleida Jeannette Flores-Pérez, Santiago Loya-López, Arturo Ávalos-Fuentes, Aida Calderon-Rivera, Elisa Damo, Fernando Lazcano-Pérez, Rajesh Khanna, Benjamin Florán-Garduño, Judith Sánchez-Rodríguez

**Affiliations:** 1 Posgrado en Ciencias del Mar y Limnología, Universidad Nacional Autónoma de México, Circuito Exterior S/N, Ciudad Universitaria, Coyoacán 04510, Mexico; alejean@comunidad.unam.mx; 2 Unidad Académica de Sistemas Arrecifales Puerto Morelos, Instituto de Ciencias del Mar y Limnología, Universidad Nacional Autónoma de México, Prolongación Niños Héroes s/n, Domicilio Conocido, Puerto Morelos 77580, Mexico; ferlaz@comunidad.unam.mx; 3 Department of Pharmacology and Therapeutics, University of Florida, 1149 Newell Drive, Gainesville, FL 32610, USA; loyalopezs@ufl.edu (S.L.-L.); aida.calderonriv@ufl.edu (A.C.-R.); r.khanna@ufl.edu (R.K.); 4 Departamento de Fisiología, Biofísica y Neurociencias, Centro de Investigación y de Estudios Avanzados del Instituto Politécnico Nacional, Av. IPN 2508, Alcaldía Gustavo A. Madero, Mexico City 07360, Mexico; javalos@cinvestav.mx (A.Á.-F.); bfloran@fisio.cinvestav.mx (B.F.-G.); 5 Department of Molecular Pathobiology, College of Dentistry, New York University, New York, NY 10010, USA; ed2671@nyu.edu

**Keywords:** *Bunodeopsis globulifera*, sea anemone, crude extract, toxin, voltage-gated channels

## Abstract

Sea anemones are an important source of bioactive compounds with potential pharmacological applications. Their toxins are produced and stored in organelles called nematocysts and act on specific targets, including voltage-gated ion channels. To date, sea anemone toxins have demonstrated effects on voltage-gated sodium and potassium channels, facilitating investigations into the structure and function of these proteins. In this study, we evaluated the effect of *Bunodeopsis globulifera* sea anemone crude extract, and of a low molecular weight fraction, on voltage-gated sodium and calcium channels within the murine nervous system. Notably, the crude extract led to a significant reduction in total sodium current, while also triggering calcium-dependent glutamate release. Furthermore, the low molecular weight fraction, in particular, enhanced total calcium currents and current density. These findings underscore the existence of sea anemone toxins with diverse mechanisms of action beyond those previously documented.

## 1. Introduction

The study of venoms is of particular interest in a variety of fields, including evolutionary ecology and biotechnology because they are an important source of bioactive compounds [1]. The bioactive constituents of venoms, often referred to as toxins, either individually or synergistically target signaling cascades with remarkable affinity and potency [2]. Toxins from marine invertebrates have garnered special attention in drug discovery because they display activities that support a wide spectrum of pharmacological and medical applications [3]. For instance, Prialt^®^, approved by the FDA in 2004, is used in the clinic as a potent analgesic; its active ingredient, called Ziconotide, is a synthetic version of ω-conotoxin MVIIA, obtained from the marine mollusk *Conus magus* [4,5]. Various marine phyla have been studied to extract and refine compounds for nutritional or medical applications. Among these, the phylum Cnidaria, which includes sea anemones, corals, jellyfish, and hydrozoans, consists exclusively of venomous animals [6]. These organisms possess a unique venom delivery mechanism called nematocysts, which are located on several sites depending on the species. For instance, in Scyphomedusa, nematocysts are distributed in the tentacles, endoderm, and gastric cirri, whereas in sea anemones can be well distributed along the whole body [7]. The toxins can also be dispersed in the mucus coating that envelopes the body of the organism in order to help their defense and protect from infections [8,9]. Nematocysts carry a cocktail of toxins for both predation and defense [10]. These toxins contain a mixture of compounds of proteinaceous (e.g., peptides, proteins and enzymes) and non-proteinaceous (e.g., purines, quaternary ammonium compounds, biogenic amines, and betaines) nature [11,12]. When exposed to these toxins, individuals may experience anything from minor local irritation to severe reactions, including excruciating pain and life-threatening cardiovascular collapse. These effects arise due to the interactions of toxins with ion channels in both the Central Nervous System (CNS) and the neuromuscular junction [10,13] and, in the case of dangerous jellyfishes and box jellyfishes, by cytolysins that destroy erythrocytes, releasing massive amounts of potassium into the bloodstream [14].

Sea anemone neurotoxins are low molecular weight peptides with toxic activity mostly due to their effects on a swath of ion channels. The best known of these neurotoxins are the peptides isolated from sea anemones, which display effects on ion channels. For instance, the peptide APETx2, isolated from *Anthopleura elegantissima*, reversibly inhibits the Acid Sensing Ion Channel 3 (ASIC3) [15]. Gigantoxin-I, isolated from *Stichodactyla gigantea*, indirectly activates Transient Receptor Potential Vanilloid 1 (TRPV1) channels when co-applied with capsaicin [16]. Recently, ShK, a potent K_v_ channel toxin isolated from *Stychodactyla helianthus* sea anemone, was proposed as a potential therapeutic for multiple sclerosis treatment due to its potent block of lymphocytic K_v_1.3 channels [9]. Other type of cnidarians, such as zoanthids, were also explored in order to find molecules with ion channel activity. The venom from *Palythoa caribaeorum*, a zoanthid, was reported to affect both voltage-gated sodium and potassium channels from rat Superior Cervical Ganglia (SCGs) by delaying the inactivation kinetics and decreasing the transient (I_A_) and delayed rectifier (I_DR_) current, respectively [17]. This same venom also decreased the current density through the N-type voltage-gated calcium (Ca_V_2.2) channels in rat SCGs [17], a channel targeted by ω-conotoxin MVIIA to block pain.

*Bunodeopsis globulifera*, a sea anemone inhabiting the Mexican Caribbean Sea, is notorious for its severe stinging due to the toxins it harbors [18]. Although cytotoxic effects on a lung adenocarcinoma cell line were reported in 2013 [19], the precise mechanism and properties of these anemone toxins remain insufficiently explored. In our study, we unveil the first insights into this mechanism of action on both the central and peripheral nervous systems. Notably, Matrix-Assisted Laser Desorption Ionization Time-of-Flight (MALDI-TOF-MS) spectrometry analysis revealed the presence of low molecular weight compounds. We assessed the effect of these compounds on glutamate release in the rat cortex and on voltage-gated sodium and calcium channels in the rat Dorsal Root Ganglion (DRG) using whole-cell patch-clamp electrophysiology. Here, we demonstrate that *B. globulifera* low molecular weight compounds: (1) promote an increase of basal and KCl-evoked glutamate release; (2) decrease Na^+^ currents and modify the inactivation kinetics of sodium channels; and (3) increase Ca^2+^ total currents, also modifying their kinetics of inactivation. Together, these results provide instructive clues as to how *B. globulifera* toxins affect the vertebrate nervous system.

## 2. Results and Discussion

Sea anemones, like other venomous animals, produce complex mixtures of bioactive molecules. Some of them are proteinaceous (e.g., peptides, proteins, and enzymes) and others are non-proteinaceous compounds of low molecular weight (e.g., purines, polyamines, and salts), and are known to modulate or selectively act on both sodium and potassium voltage-gated ion channels. Although sea anemone toxins have been studied as molecular tools and potential therapeutic agents, the isolation and functional identification of distinct peptides remains a challenge [12,20,21].

### 2.1. Crude Extract and F1 Bioactivity

**Figure 1:**
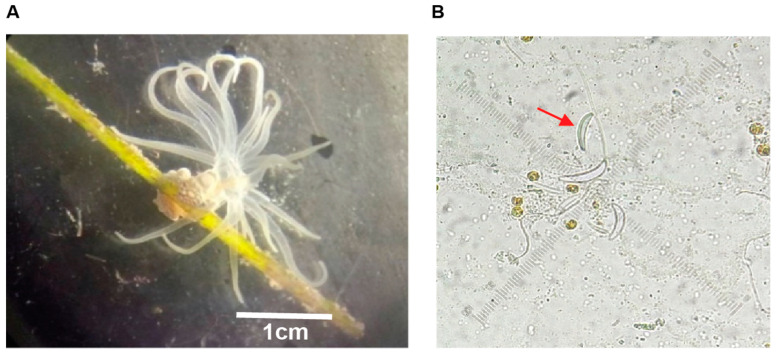
*B. globulifera* sea anemone and its nematocyst. (**A**): *B. globulifera* (Boloceroididae family and Anthozoa class) is a sea anemone associated with seagrasses in the Caribbean Sea, with size between 2 and 3 cm approximately. Sea anemones have toxins in their nematocyst, capsule-like structures. Basitric-isorhiza is one of the nematocysts type present in *B. globulifera*. (**B**): A group of nematocysts is shown, where the toxins have already been discharged by maceration, seen under microscope (40×). The red arrow shows an undischarged nematocyst.

**Figure 2:**
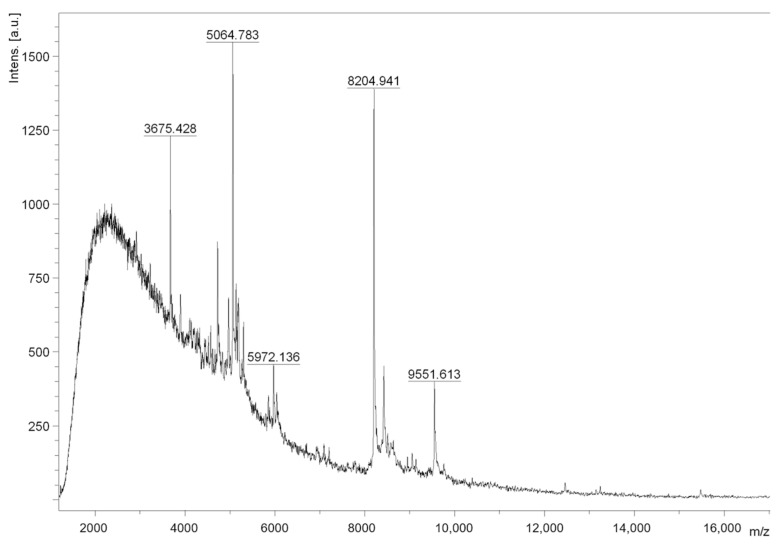
MALDI-TOF-MS spectrum from fraction F1 obtained from *B. globulifera*. This fraction showed compounds between 3.6 and 9.5 kDa.

During the extraction process of the toxins from *B. globulifera* (Figure 1A), nematocysts were monitored. The presence of basitric-isorhiza and microbasic mastigophore types were observed (Figure 1B) and the taxonomy of the species was confirmed as described in González-Muñoz [22]. The protein concentration of CE was 0.065 mg protein/mg extract (6.5% protein) and F1 protein concentration was 0.089 mg protein/mg F1 (8.9% protein). In order to qualitatively assess neurotoxicity, symptoms were observed with the injection (50 µL) of Crude Extract (CE) at 5 mg/mL to *Ocypode quadrata* crabs (*n* = 3). CE elicited intense tremors, uncontrolled movements in the legs and eyes, followed by paralysis and death. Crabs injected with deionized water (control) did not present any untoward symptoms. Fraction F1, obtained by ultrafiltration of CE, was also tested in crabs (*n* = 3); however, the symptoms were observed at a lower concentration injection (50 µL F1 [1 mg/mL]) and caused spasms, intense tremors for long periods (hours), paralysis, and death. These effects were similar to the neuromuscular system alterations previously reported [23]. MALDI-TOF mass spectrometry analysis of fraction F1 confirmed the presence of compounds between 3.6 and 9.5 kDa (Figure 2). This result is consistent with molecular weights reported for neurotoxins identified from sea anemones. Neurotoxins acting on Voltage-Gated Sodium Channels (VGSCs) have masses between 3 and 5 kDa, whereas neurotoxins acting on voltage-gated potassium channels (VGKCs) have a mass between 3.5 and 6.5 kDa [13].

### 2.2. Neurotoxic Activity

The principal function of toxins is to immobilize their prey or to survive predation. The targets of these toxins include voltage-gated sodium, calcium, and potassium ion channels which are involved in action potential generation and neurotransmission in excitable cells [24]. Cnidarian neurotoxins, particularly those isolated from sea anemones, modulate ion channels in excitable cells [3,20]. Here, we report that the crude extract and fraction F1 from *B. globulifera* cause paralysis and motor dysfunction in ghost crabs, a helpful assessment for monitoring neurotoxic activity. These neurotoxins are also active on mammalian voltage-gated channels and are considered potential pharmacological tools. Since motor control in humans is controlled by the central nervous system, and in order to identify a molecular target or possible mechanism of action of CE and F1, we analyzed both samples on Central and Peripheral Nervous System tissues (CNS and PNS).

#### 2.2.1. Effect of *B. globulifera* CE on Cortical [^3^H]-Glutamate Release

**Figure 3:**
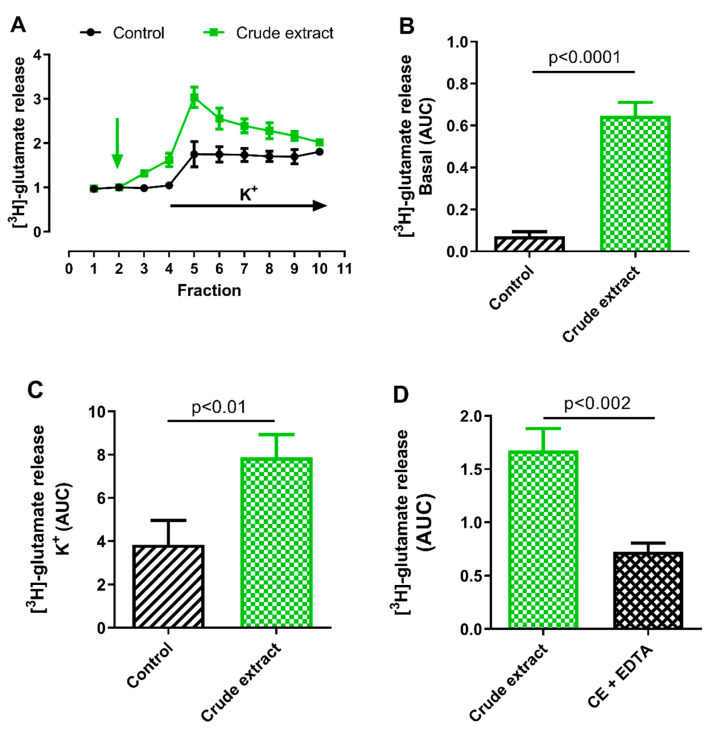
Crude extract from *B. globulifera* increases [^3^H]-glutamate release in rat cortex slices. (**A**): A typical experiment of the fractional course of the K^+^- induced [^3^H]-glutamate release in cortex slices. [^3^H]-glutamate release in control (black circles) or in the presence of crude extract (CE; 50 μg of protein/mL; green squares) is shown. The vertical arrow indicates the initiation of the CE perfusion, and the horizontal arrow, the initiation of perfusion with K^+^ (30 mM). Bar graphs showing the change depicted in the relative Area Under the Curve (AUC) for CE and control. (**B**): Basal glutamate release caused by CE. (**C**): Glutamate release in presence of CE and a depolarizing pulse. (**D**): Glutamate release in presence of CE and EDTA. The decrease in the release of glutamate in the presence of CE+EDTA, suggests the participation of calcium in the effects caused by CE alone. Student’s *t*-test. *n* = 3 experiments, five replicates per experiment.

We first evaluated the effect of CE on glutamate release in rat cortical slices. CE (50 μg of protein/mL) was applied by perfusion to rat cortex slices and radioactive glutamate release was quantified. CE significantly increased basal [^3^H]-glutamate release by 795% compared to the control (*p* < 0.0001; *n* = 3; Figure 3A,B). Moreover, when depolarizing stimulus (KCl 30 mM) was applied, the glutamate release induced by CE was 119% higher compared to the control (*p* < 0.01; *n* = 3, Figure 3C). Interestingly, both results show that CE stimulates the release of glutamate in CNS.

Neurotransmitter release depends both on extracellular calcium influx through Voltage-Gated Calcium Channels (VGCCs) and from internal calcium reservoirs. In order to identify whether the effect of CE was due to a mechanism related to calcium, the basal release of [^3^H]-glutamate was evaluated in the presence of the calcium chelator EDTA (1 mM). As shown in Figure 3D, [^3^H]-glutamate release was inhibited by ~60% when EDTA was added (*p* < 0.002; *n* = 4), indicating that the glutamate release stimulated by CE might involve a calcium-dependent mechanism. In the brain, the main excitatory neurotransmitter is glutamate, which causes excitotoxicity and neurodegeneration at high concentrations, including epilepsy [25]. The significant increase of the release of glutamate promoted by CE can therefore cause toxicity to the CNS and affect or interfere with the proper functioning of the cortex, which is associated with the generation of motor responses. It is hypothesized that in the muscles of crustaceans, glutamate operates at the neuromuscular junction [26]. This corresponds with our findings in crabs, which exhibited uncontrolled movements following administration of CE, presumably due to the neurotoxicity of glutamate. Over the past several decades, various impacts of sea anemone venoms on glutamate have been documented. It was reported that the application of fraction D, derived from the sea anemone *Actinostella flosculifera* (Le Sueur, 1817) = (*Phyllactis flosculifera*), led to a reduction in the glutamatergic response (at concentrations of 2–8 mg/mL) in neurons of *Z. guanensis* [27]. This effect was found to be dose-dependent and could be partially reversed within approximately 30 min after washing. The researchers reported that the exudate from this sea anemone exhibits an antiglutamatergic property. In another study [28], it was observed that the Bc2 fraction derived from the sea anemone *Bunodosoma caissarum* triggered the release of synaptosomal glutamate, a process that was independent of extracellular Ca^2+^ and did not involve voltage-sensitive Na^+^ channels. They hypothesized that this could be due to a direct action similar to α-Latrotoxin (LTX), a potent toxin from black widow spider venom that induces a robust release of neurotransmitters by acting on synaptotagmin. An alternate theory proposed was that Bc2 directly interacts with the exocytotic machinery.

Another study with *B. caissarum* reported that the crude extract obtained from this organism causes dose-dependent convulsions in mice when administered by the i.c.v. route [29]. They also observed that the crude extract inhibited glutamate binding to cerebral cortical membranes and enhanced glutamate release from cortical synaptosomes. This group concluded that the convulsions elicited are not due to the presence of cytolysin or related to an increase in glutamate release but may depend on the interaction between a peptide component of the extract and NMDA receptors.

**Figure 4:**
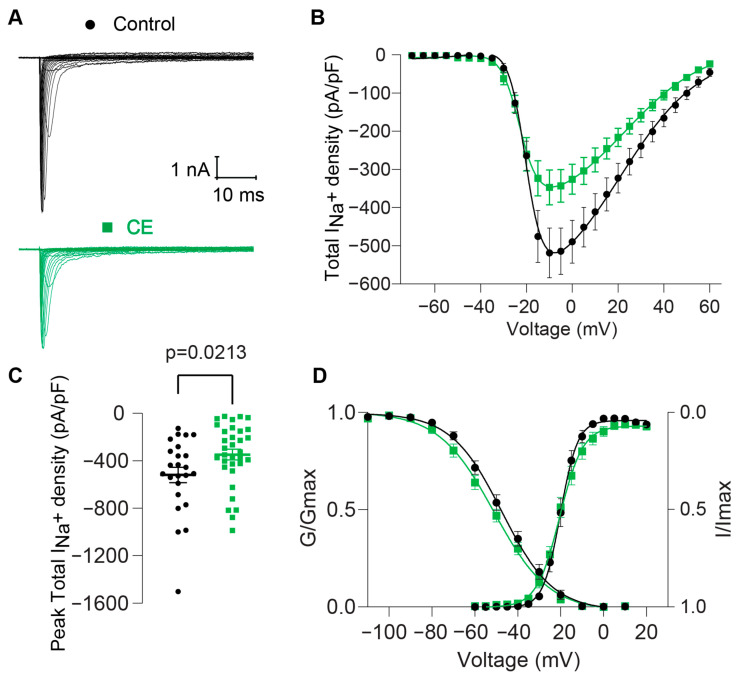
The Crude Extract (CE) from *B. globulifera* decreases total sodium current (I_Na_^+^) in dorsal root ganglion neurons (DRGs) and modifies the inactivation kinetics of sodium channels. Control: black circles, CE: green squares. The CE [50 μg of protein/mL] decreases the density of the total Na^+^ current (I_Na_^+^) recorded from rat DRGs. (**A**): Representative traces of I_Na_^+^, control and in the presence of CE. (**B**): Summary of I_Na_^+^ density vs. voltage relationship. (**C**): Bar graphs of total peak I_Na_^+^ density. Significant differences were observed in the I_Na_^+^ density from CE, compared with the control. (**D**): Boltzmann fits for normalized conductance voltage relationship for voltage-dependent activation (G/Gmax) and inactivation (I/Imax). Significant differences were observed in the V_1/2inact_ between groups. Error bars indicate mean ± SEM; *p* = 0.03; *n* = 24 (control), *n* = 33 (CE).

Our findings partially align with the study conducted by Migues et al. [28], who reported an increase in glutamate release upon the application of sea anemone venom. However, we discovered that the use of a calcium chelator from the extracellular medium led to a 60% reduction in the response triggered by *B. globulifera* (Figure 4D). This suggests that the toxins present in *B. globulifera* may operate through a calcium-dependent mechanism.

#### 2.2.2. Effect of *B. globulifera* CE on Voltage-Gated Sodium Channels of Rat DRG Sensory Neurons

Voltage-Gated Sodium Channels (VGSCs) are targets of over 50 sea anemone toxins [30]; however, the effects of the toxins from *B. globulifera* on VGSCs are unexplored. To assess the effect of CE [50 μg of protein/mL] on VGSCs, we recorded total sodium currents from rat DRG sensory neurons through patch voltage-clamp electrophysiology. Large transient inward currents were observed in response to depolarizing voltage steps (Figure 4A). The mean peak sodium current density (at −10 mV) of the control condition was −518.81 ± 65.3 pA/pF (control, *n* = 24, Figure 4B,C). The presence of CE in the external recording solution significantly decreased total Na^+^ current and current density, promoting a 33% decrease (−347.18 ± 45.65 pA/pF, *n* = 33, Figure 4A–C) compared to the control condition. Analysis of voltage-dependence inactivation revealed a significant ~4 mV shift toward more negative voltages (Figure 4D).

These findings provide novel evidence that the low molecular weight peptides present in the CE from *B. globulifera* exert activity on sodium channels.

Sodium channel toxins are some of the best characterized cnidarian toxins. They bind to (extracellular) loop S3-S4 in domain IV of Na^+^ channels, locking the S4 segment in its inward position and thus inhibiting conformational changes for channel fast inactivation [31,32]. All of these toxins were sequenced and shown to delay sodium channel inactivation. Even total venoms tested on this channel induce this delay on the inactivation [30,33]. However, our experiments showed a completely different result: the existence of toxins in *B. globulifera* crude extract that do not delay the channel inactivation nor cause an increase in the total sodium current. Instead, the crude extract decreased the total Na^+^ current, suggesting that there might be a component or components that inhibit this channel. This was also observed for the venom of another sea anemone, *Bartholomea annulata* [12].

Our results suggest that the toxins produced by *B. globulifera* may directly interact with VGSCs, altering their function and contributing to the toxic effects observed in other organisms exposed to them. The leftward shift on the inactivation kinetics indicates that the sodium channels are more refractory to opening, likely due to the interaction of one or several compounds in the crude extract with voltage regulating domains, which might be ultimately leading to a decreased ion influx through them, reflected as the decrease in the current density that we observed. The present work contributes to our understanding of the molecular mechanisms underlying the toxic effects of sea anemone toxins and highlights the potential of these toxins as pharmacological tools for studying VGSC function. Further investigation into the specific sodium channel subtypes and mechanisms of action of the toxins from *B. globulifera* could provide valuable insights for drug discovery and development efforts.

#### 2.2.3. Effect of *B. globulifera* CE and F1 in Voltage-Gated Calcium Channels of Rat DRG Sensory Neurons

**Figure 5:**
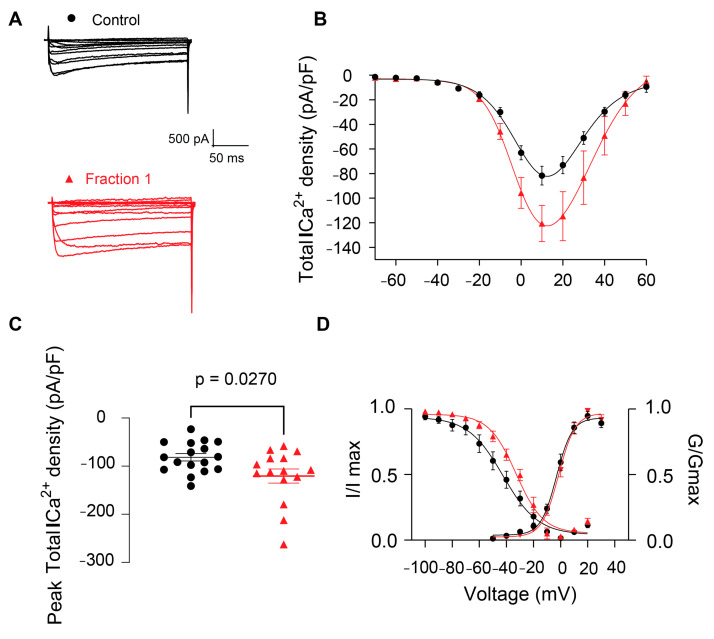
Fraction F1 from *B. globulifera* increases total calcium currents (I_Ca_^2+^) in dorsal root ganglion neurons (DRGs) and modifies the inactivation kinetics of calcium channels. Control: black circles, Fraction F1: red triangles. The fraction F1 [75 μg of protein/mL] increases the density of the total calcium currents (I_Ca_^2+^) recorded from rat DRGs. (**A**): Representative traces of I_Ca_^2+^, control and in the presence of fraction F1. (**B**): Summary of I_Ca_^2+^ density vs. voltage relationship. (**C**): Bar graphs of total peak I_Ca_^2+^ density. Significant differences were observed in the I_Ca_^2+^ density from fraction, compared with the control. (**D**): Boltzmann fits for normalized conductance voltage relationship for voltage-dependent activation (G/Gmax) and inactivation (I/Imax). Significant differences were observed in the V_1/2inact_ of fraction F1, when compared with the control. Error bars indicate mean ± SEM; *p* = 0.02; *n* = 17 (control), *n* = 15 (fraction).

Given the observed increase in radioactive glutamate release with CE and the well-known coupling between release and calcium dependency, we next proceeded to examine whether the effect of CE in neurotransmission was due to an effect on VGCCs. Ba^2+^ mediated the inward currents, denoted as Ca^2+^ currents, which were elicited by depolarizing voltage steps (Figure 5A). The mean peak total calcium current density (taken at +10 mV) of the control condition was −81.66 ± 7.64 pA/pF (control, *n* = 17, Figure 5B,C). The presence of CE [50 μg of protein/mL] in the external solution did not allow us to get successful electrophysiological recordings, possibly indicating interference or perturbation to the membrane or to the external solution osmolarity caused by the extract. For this reason, we then tested F1 [75 μg of protein/mL], which also showed biological activity when administered to *O. quadrata* crabs. The addition of F1 to the external recording solution significantly increased total Ca^2+^ currents and current density, promoting a ~47% increase (−120.62 ± 14.69 pA/pF, *n* = 15, Figure 5A–C) with respect to the control condition. Analysis of voltage-dependence inactivation revealed a significant ~10-mV shift toward more positive voltages (Figure 5D). Our results provide novel insights into the mechanism of action of fraction F1 and its effects on VGCCs and subsequent neurotransmitter release facilitation. The augmented current density could be most likely explained by the markedly significant rightward shift in the inactivation kinetics, which would render the calcium channels more prone to remaining active. We hypothesize that F1 might be directly binding to the voltage-sensing domain of VGCCs, leading to an increased influx of calcium through these channels.

To date, very few works have assessed the effect of some cnidarian venoms on calcium channels [17,20,34,35]. Our results showed that *B. globulifera* fraction (F1) increases the total Ca^2+^ current, suggesting an agonistic effect. This is surprising, since there are very few compounds known to date that can elicit this type of activity: the cyclodepsipeptide aplidine isolated from the tunicate *Aplidium albicans* (L-type calcium channel enhancer), maitotoxin from *Gambierdiscus toxicus* (L-type calcium channel activator), and yessotoxin, which produces Ca^2+^ influx through nifedipine and SKF-sensitive channels [36]. The only similar report for another cnidarian is the work by Qar and collaborators [34] that describes a 19 kDa protein from a scleractinian coral that is a calcium channel activator.

Further investigation into the specific peptides responsible for these effects and their precise mode of interaction with VGCCs could elucidate the underlying molecular mechanisms and potentially uncover therapeutic applications or pharmacological targets. Ca_V_2.2, a very abundant calcium channel subtype in DRGs, might be one of the channel targets for this effect, since it has already been reported that cnidarian toxins can target these channels in rat SCGs, although by inhibiting them [17].

## 3. Materials and Methods

### 3.1. Sea Anemone Collection

*B. globulifera* was collected by scuba diving at approximately 2 m depth in the reef lagoon of Puerto Morelos, Q. Roo, Mexico. The organisms were transported to the laboratory inside hermetic sealed plastic bags containing sea water. Once in the laboratory, the sea anemones were placed in glass vials containing 2 mL of deionized water and stored at −60 °C.

### 3.2. Crude Extract Obtention

The organisms were thawed and macerated with a glass homogenizer (Tenbroeck Pyrex^®^, Corning Inc., Corning, NY, USA). Nematocyst discharge was monitored with a microscope in order to ensure the obtention of the active compounds. The sample was then centrifuged at 3200× *g* for 30 min at 4 °C. The supernatant was recovered and placed in 50 mL conical tubes (Falcon^®^ Corning Inc., Corning, NY, USA). The tubes were frozen at −60 °C, lyophilized in a freeze-drying equipment (Labconco^®^ 77500, Kansas City, MO, USA) and stored at −70 °C.

### 3.3. Protein Quantification

Proteins were quantified by the Bradford method [37] with the Assay Bio-Rad^®^ Quick Start kit (Hercules, CA, USA) Bovine Serum Albumin (BSA) was used as standard. The absorbance was monitored at 595 nm in a microplate reader (Stat Fax^®^ 4200, Awareless, Palm City, FL, USA).

### 3.4. Biological Activity Assay

The toxic activity of the extract and fractions was tested using the crab bioassay proposed by Béress and Béress [38]. Crabs of the species *Ocypode quadrata* were collected at the beach and injected with 50 µL of CE (5 mg/mL) into the thorax–coxa junction of the third walking appendage. Deionized water was used as vehicle. The symptoms were observed and recorded.

### 3.5. Crude Extract Fractionation and Fraction F1 Mass Spectrometry Analysis

The CE was separated using an Amicon^®^ system (Millipore^®^ Merck, Darmstadt, Germany) with 10 kDa molecular weight cut-off membrane. The <10 kDa fraction (F1) was collected, lyophilized, and stored at −60 °C. The F1 was tested in crabs and the molecular mass range of the F1 components was determined by MALDI-TOF mass spectrometry. 5 µL of a saturated solution of sinapinic acid were added to 5 µg of lyophilized sample. 1 µL of this solution was deposited onto the MALDI plate and allowed to dry at room temperature. The spectrum was recorded on linear positive mode on a mass spectrometer Microflex Bruker Daltonics, equipped with nitrogen laser at 337 nm.

### 3.6. Neurotoxic Activity Assays

#### 3.6.1. [^3^H]-Glutamate Release

The experiments were done using a method adapted from Briones–Lizardi et al. [39]. Male Wistar rats maintained and handled according to the guidelines of the CINVESTAV-IPN Animal Care Committee (Reference 0146-15) were euthanized and the brains isolated and immersed in oxygenated ice-cold Artificial Cerebro Spinal Fluid (ACSF) (solution composition (mM): NaCl 118.25; KCl 1.75; MgSO_4_ 1; KH_2_PO_4_ 1.25; NaHCO_3_ 25; CaCl_2_ 24; D-glucose 10; pH 7.4; bubbled with O_2_/CO_2_ 95:5 *v*/*v*) and coronal sections (300 µm thick) were made with a vibroslicer CAMPDEN Instruments Ltd., the cortex was identified based on the atlas Paxinos and Watson (1997) [40], and was microdissected. The slices obtained and pooled in a single incubation assay tube were allowed equilibrate in ACSF (30 min at 37 °C, gassed with O_2_/CO_2_ 95:5 *v*/*v*).

##### Radioactive Labeling with [^3^H]-Glutamate

After the stabilization time, the slices were incubated in 2 mL for 30 min with ACSF that contained [^3^H]-glutamate (95 Ci/mmol) 100 nM, aminooxyacetic acid (to inhibit glutamate decarboxylase and prevent the conversion of glutamate to GABA), and 200 µM dihydrokainic acid to prevent the uptake of [^3^H]-glutamate by astrocytes.

##### Experimental Protocol

The slices were placed in perfusion chambers and superfused with ACSF at a rate of 0.5 mL/min in a constant temperature bath maintained at 37 °C. Each chamber (volume: 80 µL) contained five or six slices; five chambers of the superfusion apparatus conformed an experimental group. Experiments were reproduced three times. In order to remove the [^3^H]-glutamate trapped in the interstitial space, the slices were superfused with normal ACSF for 20 min before collecting the fractions for counting the radio activity. With a fraction collector, superfused fractions were obtained every 4 min in a volume of 2 mL. For control group (basal condition release), four fractions were first collected, then [K^+^] in the ACSF was increased to 30 mM (solution composition (mM): NaCl 106.25, KCl 28.75, MgSO_4_1, KH_2_PO_4_ 1.25, CaCl_2_ 2, NaHCO_3_ 25, D-glucose 10, pH 7.4) and six fractions were collected (high K^+^ medium); for CE group, CE obtained from *B. globulifera* [50 µg of protein/mL] was added to the medium after one fraction to record the effects on release in both basal and high K^+^ condition.

For the EDTA group, first three fractions of basal release were collected, next the CE was added and three fractions were collected. Finally, EDTA [1 mM] was added to chelate calcium in the medium and record whether the [^3^H]-glutamate release is calcium dependent. Six fractions were collected. The experiments were reproduced four times.

##### Quantification of [^3^H]-Glutamate Release

Liquid scintillation counting was used for measured the radioactivity released into the superfusion medium in each fraction. The slices were collected and treated with 1 mL of HCl 1M for 5 h, then scintillation liquid was added and radioactivity was quantified using a scintillation counter to determine the total amount of tritium remaining in the tissue. [^3^H]-glutamate release was expressed as a fraction of the total amount of tritium remaining in the tissue when collecting the fraction. The effect of CE on the basal release of [^3^H]-glutamate was assessed by comparing the fractional release in Fraction 1, immediately before exposure of the tissue to the CE, and Fraction 4, immediately before exposure to high K^+^ medium. Changes in K^+^ evoked [^3^H]-glutamate release were assessed by comparing the area under the curves between the first and last fractions collected after the change to high K^+^.

##### Statistical Analysis

The curves obtained in each of the [^3^H]-glutamate release experiments were analyzed with GraphPad Prism (Version 8) software to obtain the Area Under the Curve (AUC). The areas obtained for each experimental condition were compared using Student’s *t*-test. The value of *p* < 0.05 was taken as the minimum value of acceptable significance between groups.

#### 3.6.2. Electrophysiology

##### Animals

Adult female and male 150–200 g Sprague–Dawley rats were handled in accordance with the Guide for Care and Use of Laboratory Animals (published by the National Institutes of Health). The animals were housed at 23 ± 3 °C in light-controlled rooms with rodent chow and water ad libitum. All experiments performed were approved by the Institutional Animal Care and Use Committee of New York University (Reference PROTO202100104).

##### Isolation and Culture of Rat Dorsal Root Ganglion Neurons

Rats were euthanized with isoflurane and subsequently decapitated. A laminectomy was carried out and thoracic and lumbar Dorsal Root Ganglia (DRGs) were excised and placed in sterile DMEM (Thermo Fisher Scientific, Waltham, MA, USA; Cat# 11965) in combination with 5 mg/mL type I collagenase, (Cat# LS004194, Worthington, DC, USA) and 3.125 mg/mL of neutral protease (Cat# LS02104, Worthington, Lakewood, NJ, USA) for 50 min at 37 °C under gentle agitation. The dissociated cells were then centrifuged at 800 rpm for 7 min and resuspended in DMEM containing 1% penicillin/streptomycin sulfate (Life Technologies, Carlsbad, CA, USA; Cat# 15140), 10% fetal bovine serum (HyClone). The cells were seeded on 0.1 mg/mL poly-D-lysine (Millipore Sigma, St. Louis, MO, USA; Cat# P6407) and 1 mg/mL laminin (Santa Cruz Biotechnology, Dallas, TX, USA; Cat#sc-29012) -coated 12-mm glass coverslips and incubated at 37 °C.

##### Patch-Clamp Electrophysiology

Experiments were performed using a method adapted from Loya-Lopez et al. [41]. Whole-cell voltage-clamp recordings were carried out 12–18 h after culture by using an EPC 10 HEKA amplifier. Total sodium (Na^+^) currents were recorded in the absence or presence of CE (50 µg of protein/mL) in the external recording solution. On the other hand, calcium (Ca^2+^) currents were recorded in the absence or presence of either CE (50 µg of protein/mL) or the F1 fraction (75 µg of protein/mL) in the external recording solution.

##### Voltage-Clamp Recordings

DRG neurons were subjected to current-voltage (I-V) and activation or inactivation voltage protocols as follows: for total sodium currents (I_Na_^+^), cells were held at a potential of −60 mV and depolarized by 150-ms voltage steps from −70 mV to 160 mV in 5-mV increments.

The external solution contained in mM: NaCl 140, tetraethylammonium chloride (TEA-Cl) 30, D-glucose 10, KCl 3, CaCl_2_ 1, CdCl_2_ 0.5, MgCl_2_ 1, and HEPES (pH 7.3 and 310–315 mOsm) 10; patch pipettes were filled with an internal solution containing (in mM): CsF 140, NaCl 10, Cs-EGTA 1.1, and HEPES (pH 7.3 and 290–310 mOsm/L) 15.

For total Ca^2+^ currents (I_Ca_^2+^), cells were held at a potential of −90 mV and depolarized by 200-millisecond voltage steps from −70 to +60 mV in 10-mV increments. The external solution contained (in mM): N methyl-D-glucamine 110, BaCl_2_ 10, TEA-Cl 30, HEPES 10, and glucose 10 (pH 7.29 adjusted with TEA-OH, and 310–315 mOsm/L). Patch pipettes were filled with an internal solution containing in mM: CsCl_2_ 150, HEPES 10, Mg-ATP 5, and BAPTA 5, (pH 7.24 adjusted with CsOH, and 290–305 mOsm/L).

Normalization of currents to each cell’s capacitance (pF) was performed to allow for collection of current density data. For I-V curves, functions were fitted to data using a non-linear least squares analysis. I-V curves were fitted using double Boltzmann functions:

f = a + g1/(1 + exp((x − V_1/2_1/k1)) + g2/(1 + exp ( − (x − V_1/2_2/k2))

where x is the prepulse potential, V_1/2_ is the midpoint potential, and k is the corresponding slope factor for single Boltzmann functions.

Double Boltzmann fits were used to describe the shape of the curves, not to imply the existence of separate channel populations. The numbers 1 and 2 simply indicate the first and second midpoints; *a* along with *g* are fitting parameters.

Activation curves were obtained from the I-V curves by dividing the peak current at each depolarizing step by the driving force according to the equation: G = I/(V_mem_ − E_rev_), where I is the peak current, V_mem_ is the membrane potential, and E_rev_ is the reversal potential. The conductance (G) was normalized against the maximum conductance (G_max_). For total Ca^2+^ currents, Steady-State Inactivation (SSI) curves were obtained by applying an H-infinity protocol that consisted of 1.5-s conditioning pre-pulses from −100 to +30 mV in 10-mV increments followed by a 20-ms test pulse to +10 mV. For Na^+^ currents, SSI curves were obtained by applying an H-infinity protocol that consisted of 1-s conditioning pre-pulses from −120 to +10 mV in 10-mV increments followed by a 200-ms test pulse to +10 mV.

Inactivation curves were obtained by dividing the peak current recorded at the test pulse by the maximum current (I_max_). Activation and SSI curves were fitted with the Boltzmann equation. The curves were analyzed using the Fitmaster software (v2x91, HEKA).

##### Statistical Analysis

Normalized peak currents were compared with a Mann–Whitney test. V_1/2_ midpoint potential and *k* slope factor were analyzed by an unpaired *t*-test.

## 4. Conclusions

The neurotoxic properties of toxins derived from *B. globulifera* can be explained by their interactions with ion channels involved in electrogenesis and neurotransmission. This sea anemone showed itself to be a source of toxins with new and unexplored effects.

Understanding the molecular mechanisms underlying these effects could lead to the development of novel therapeutic agents or pharmacological tools for studying neuronal function. The present study underscores the significance of toxinology in the discovery of molecules with pharmacological potential from venomous animals.

## Data Availability

Data is contained within the article.
